# Liver Lesions in Estuarine Dolphins in the Indian River Lagoon, Florida: Does Microcystin Play a Role?

**DOI:** 10.3390/toxics12120858

**Published:** 2024-11-27

**Authors:** Ami Krasner, Wendy Noke Durden, Megan Stolen, Teresa Jablonski, Agatha Fabry, Annie Page, Wendy Marks, Cecilia Costa, H. C. D. Marley, Spencer Fire

**Affiliations:** 1 Department of Ocean Engineering and Marine Sciences, Florida Institute of Technology, Melbourne, FL 32901, USAsfire@fit.edu (S.F.); 2 Hubbs-SeaWorld Research Institute, Melbourne Beach, San Diego, FL 32951, USA; wnoke@hswri.org (W.N.D.);; 3 Blue World Research Institute, Cocoa, FL 32927, USA; 4 Harbor Branch Oceanographic Institute, Florida Atlantic University, Ft. Pierce, FL 34946, USA

**Keywords:** microcystin, liver, Indian River Lagoon, sentinels, bottlenose dolphin, ELISA, MMPB, respiratory vapor

## Abstract

Microcystin (MC), a hepatotoxin produced by cyanobacteria, was introduced into the Indian River Lagoon (IRL), Florida, in 2005 through freshwater outflows. Since then, MC has been detected in humans, domestic animals, and wildlife in the lagoon. Potential public health effects associated with MC exposure along the IRL include an increased risk of non-alcoholic liver disease among area residents. Yet, there are limited studies characterizing liver disease, as well as the potential role of MC, in humans and animals in this region. Thus, histopathology reports (*n* = 133) were reviewed in the stranded common bottlenose dolphin (*Tursiops truncatus truncatus*) (*n* = 156, 2005–2024) to describe liver lesions in this important IRL sentinel. Liver and fecal samples (*n* = 161) from stranded individuals were screened for MC via an enzyme immunoassay (ELISA). These samples were then confirmed via the 2-methyl-3-methoxy-4-phenylbutyric acid technique (MMPB) to evaluate whether liver histopathologic lesions were linked to MC exposure. Minimally invasive MC screening methods were also assessed using respiratory swabs and vapor. Inflammation (24%, *n* = 32), fibrosis (23%, *n* = 31), lipidosis/vacuolation (11%, *n* = 15), and necrosis (11%, *n* = 14) were the most common liver anomalies observed. These non-specific lesions have been reported to be associated with MC exposure in numerous species in the peer-reviewed literature. Ten bottlenose dolphins tested positive for the toxin via ELISA, including two individuals with hepatic lipidosis, but none were confirmed by MMPB. Thus, this study did not provide evidence for MC-induced liver disease in IRL bottlenose dolphins. Other causes should be considered for the lesions observed (e.g., heavy metals, metabolic disease, and endoparasites). Respiratory swabs require further validation as a pre-mortem MC screening tool in free-ranging wildlife.

## 1. Introduction

Natural toxins produced by harmful algal blooms (HABs), including cyanobacteria, are increasingly documented worldwide as causative agents of illness and death in organisms that contact or consume contaminated water or prey [1,2]. Natural and anthropogenic influences contribute to a wider geographic range, increased incidence, growing intensity, and an emerging number of toxicogenic HAB species [3]. Certain aquatic megafauna are considered sentinels of biotoxin exposure and related health impacts by demonstrating potential threats to other wildlife, public health, and ecosystem health [4].

A key geographic region where aquatic megafauna may serve as sentinels of biotoxin-related threats is in the Indian River Lagoon (IRL) watershed [4,5]. The IRL is a diverse, subtropical estuarine ecosystem on the Atlantic coast of central Florida [6]. Over 4000 species of flora and fauna inhabit the lagoon [7], including commercially important fish, threatened or endangered wildlife, and sentinel species [4,5,8,9,10]. Potential sentinels of biotoxin exposure in the IRL include common bottlenose dolphins (*Tursiops truncatus truncatus*), North American river otters (*Lontra canadensis*), Florida manatees (*Trichechus manatus*), bull sharks (*Carcharhinus leucas*), and green sea turtles (*Chelonia mydas*) [5,9,10,11]. These species are ideal candidates based on their longevity, high trophic level status, susceptibility to toxin bioaccumulation and subsequent health effects, well-understood biology, capability of being researched, abundance, accessibility, estuarine residency, and/or dependency on the lagoon for survival [4,12,13,14,15,16]. Since these megafauna move throughout the ecosystem, they can complement data from stationary monitoring efforts across spatiotemporal scales [17]. Moreover, the IRL dolphin population is consistently observed and well studied due to high public and scientific interest in their welfare, thus facilitating their use as early warning signs of potential toxic threats [4,9,18]. Multiple HAB toxins, including microcystins (MCs), nodularins (NODs), domoic acid (DA), okadaic acid (OA), brevetoxin (PbTx), and saxitoxin (STX), have been identified in the body fluids or tissues of IRL sentinels [1,5,10,19,20,21,22]. While these biotoxins were associated with stranding events and mortality [21], the health impacts from exposure to IRL sentinels are often difficult to ascertain [5].

Microcystin is a liver toxin and tumor-promotor produced via *Microcystis* and other freshwater cyanobacteria [23]. Although historically found in freshwater, exposure to MC may be an emerging threat in the IRL watershed [23]. MC was initially introduced via freshwater outflows from Lake Okeechobee to the Saint Lucie Estuary (SLE) and southern IRL in 2005, and its persistence and toxicity in the system are likely due to recurring freshwater outflows, local eutrophication, toxin sedimentation, and climatic factors [5,24,25]. Microcystin was detected in IRL water samples in 2005, 2016, 2018, and 2019 at levels above the World Health Organization warning threshold for potential health impacts (>20 μg/L) [25,26,27,28,29]. The highest measurements were collected in the SLE during the 2005 rainy season [25,26,27,28,29]. In 2005 and 2016, MC was detected in the SLE up to 44,988 μg/L and 4500 μg/L, respectively [28,29]. In 2018 and 2019, MC was first identified in the northern half of the IRL with levels ranging between 0.01 and 359.7 µg/L throughout the system [25,29]. Microcystin levels in the IRL were correlated with water-quality parameters, including temperature, salinity, oxygen saturation, and total dissolved nitrogen and phosphorous [25]. Though seven variants (-LR, -LA, -WR, -RR, -LF, -LY, and [DAsp3]-LR) have been identified in the IRL, the most toxic form (MC-LR) was the most abundant [28].

Microcystin has been detected in humans, pets, and wildlife residing within the IRL. In 2018, MC exposure to the public was investigated by swabbing the nasal mucosa of over 120 volunteers [29]. While MC was detected via an enzyme-linked immunosorbent assay (ELISA) in over 90% of study participants [29], confirmatory testing and clinical impact screening were not reported. Potential health effects from MC exposure along the IRL include an increased risk of non-alcoholic liver disease among area residents [30], as well as cytotoxicity in engineered mammalian and human cell lines [25,31]. Microcystin exposure was confirmed by ultra-performance liquid chromatography/tandem mass spectrometry (LC-MS/MS) in the gut, liver, and plasma of free-ranging IRL bull sharks from 2018 to 2020 [5]. The sharks were putatively healthy, but screening for MC-induced clinical impacts was not performed [5]. Microcystin exposure was also confirmed via inductively coupled plasma mass spectrometry (ICP-MS) in the liver and kidney of stranded IRL green sea turtles between 2015 and 2020 [10]. However, MC-induced histologic lesions were not apparent, and exposure was unlikely to be the primary cause of stranding [10]. Notably, MC contamination of the SLE caused acute toxicosis in six domestic dogs (*Canis lupus familiaris*) during the summer of 2018 [23]. These cases were diagnosed using several complimentary MC detection methods, including the 2-methyl-3-methoxy-4-phenylbutyric acid technique (MMPB) and ELISA, along with supportive clinical signs (vomiting, diarrhea, and lethargy), histopathology (massive hepatocellular necrosis), and serum biochemistry findings (abnormal liver enzymes, bilirubin, glucose, and albumin) [23,32]. Despite the research efforts outlined above, there is no consistent screening for MC exposure and related health impacts in IRL area residents, domestic animals, or free-ranging wildlife. This substantial knowledge gap is particularly troublesome, considering the potential for repetitive exposure to multiple HAB toxins in the system [5,10].

The objective of this study was to describe liver lesions, as well as evaluate the potential role of MC, in an IRL sentinel species. Furthermore, MC exposure via respiratory fluid and vapor was evaluated. Bottlenose dolphins were selected because they are an accessible, long-lived, apex predator with life-long residency in this estuary of national significance [33,34,35].

Though numerous liver lesions were present, and MC was detected via ELISA, our data did not confirm that MC caused the liver lesions observed. Further investigation is required to confirm MC exposure to IRL bottlenose dolphins and determine the cause(s) of liver lesions in this population.

## 2. Materials and Methods

### 2.1. Study Area and Animals

Over 250 km long (902 km^2^) and ranging in width from less than 0.5 km to nearly 9 km, the IRL extends along nearly half of Florida’s east coast with a watershed that contacts six counties, from north to south: Volusia, Brevard, Indian River, St. Lucie, Martin, and Palm Beach [25,36]. The lagoon (average depth ~1.5 m) comprises three connected estuaries: the Banana River, Indian River, and Mosquito Lagoon, as well as several tributaries [36]. The watershed’s southern boundary is Jupiter Inlet, and the northern boundary is Ponce de Leon Inlet, with a planned northern extension including the Halifax River up to High Bridge Rd. in Volusia County [37]. Live-stranded and deceased bottlenose dolphins found within the IRL watershed (northern limit: Ponce de Leon Inlet–Spruce Creek) were considered for inclusion in this study. The Hubbs-SeaWorld Research Institute (HSWRI, Melbourne Beach, FL, USA) provided bottlenose dolphin samples from the northern IRL (i.e., Brevard and Volusia counties; from Sebastian Inlet north, including Spruce Creek, Mosquito Lagoon, Banana River, and the northern and north-central Indian River), and Florida Atlantic University’s Harbor Branch Oceanographic Institute (HBOI, Ft. Pierce, FL, USA) provided bottlenose dolphin samples from the southern IRL (i.e., Indian River, St. Lucie, and Martin counties; south-central and southern Indian River). Sex was determined through an examination of the genital region and/or internal reproductive tissues. Age class was categorized based on total length (TL) measurements (a straight line from the tip of the rostrum to the fluke notch) [38]: adult male (≥246 cm), adult female (≥231 cm), juvenile male (161–245 cm), juvenile female (161–230 cm), and calf (≤160 cm) [18,39]. If TL was not available, age class was not assigned. The season of stranding was defined as rainy (May–November) or dry (December–April) [25]. Both HSWRI and HBOI are members of the Southeastern United States Marine Mammal Stranding Network operating under Stranding Agreements with NOAA Fisheries and through Section 109h of the US Marine Mammal Protection Act of 1972 (MMPA 1972).

### 2.2. Sample Collection

Liver, fecal, and respiratory-swab samples were collected during the necropsy of deceased bottlenose dolphins, retrospectively and prospectively, from 2005 to 2024. Respiratory vapor samples were collected from live-stranded bottlenose dolphins prospectively in 2021 and 2023 via HSWRI. All available liver, respiratory-swab, and vapor samples were tested. Approximately 1–30 g of liver and/or feces were collected using established protocols [40]. Swabs of the upper respiratory tract were collected with a sterile cotton-tip applicator. Three exhalations of respiratory vapor were collected onto a sterile polystyrene petri dish (94 × 16 mm) with the dish held approximately 15 cm above the blowhole. Respiratory swabs and vapor samples were included in this study to evaluate aerosol droplets for MC detection in free-ranging bottlenose dolphins. Liver, fecal, and respiratory-swab samples were stored in polypropylene containers or whirl-pack bags, and petri-dish samples were secured with aluminum foil and then placed in a Ziploc bag. All samples were frozen at −20 °C or −80 °C until analysis. Sample collection, storage, and transfers were performed under established permits from HSWRI, HBOI, the Florida Institute of Technology (FIT, Melbourne, FL, USA), and GreenWater Laboratories (Palatka, FL, USA). Samples collected from code 1 to code 3 dolphins were included in this study [41]. The specimen condition was based on established protocols: code 1 (alive), code 2 (fresh dead), code 3 (moderate decomposition, organs intact) [40].

### 2.3. Microcystin Extraction and Analysis

Microcystin screening was performed at FIT via the MCs/NODs ADDA-ELISA (Abraxis©; PN 520011; Warminster, PA, USA). The original sample was extracted based on an established protocol [42]. Liver and feces were extracted by combining a 1 g homogenized sample with 4 mL of extraction/elution solvent (90% MeOH/10% acidified water; acidified water = 99.9% deionized [DI] water/0.1% trifluoroacetic acid [TFA]) in a 15 mL polypropylene centrifuge tube. This extract was vortexed for 30 s and then centrifuged at 3400× *g* for 10 min. The extract was then diluted and precipitated by combining 1 mL of the extract supernatant and 2 mL of solid-phase extraction (SPE) diluent (99.85% DI water/0.1% formic acid/0.05% TFA) in a 20 mL glass culture tube, which was then vortexed. If the combined solution turned cloudy, it was centrifuged again at 3400× *g* for 5 min. The extract was then SPE-cleaned as follows: (1) a 10 mL C18 500 mg SPE column was conditioned by passing 10 mL of MeOH, followed by 10 mL of acidified water, through the column under vacuum pressure; (2) the 3 mL of diluted extract was passed through the SPE column under vacuum pressure, and (3) the SPE cleaned extract was eluted by passing 2 mL of the extraction/elution solvent through the column under vacuum pressure. Respiratory swabs were extracted by modifying previously described methods [29,43]. Briefly, the swab was brought to room temperature and vortexed with 500 μL of MC diluent buffer for 10 min. Petri-dish samples were extracted by washing the dish with 10 mL of 100% ethanol on a plate shaker for 1 h. The extract was transferred to a 20 mL glass tube and dried under compressed air at 37 °C for 24 h. The precipitate was resuspended with 500 μL of MC diluent buffer. Positive control swabs and petri dishes were prepared utilizing known concentrations of MC standard solution. Negative controls were prepared utilizing untreated swabs and petri dishes, as well as standard 0.

Extracts were frozen at −20 °C in a 5 mL glass vial until ELISA analysis on a 96-well plate following manufacturer instructions. In addition to the six standards (0, 0.15, 0.4, 1, 2, and 5 ng/mL) and control (0.75 ± 0.185 ng/mL) included with the ELISA kit, three additional standards (0.015, 0.05, and 3.5 ng/mL) were prepared by diluting standard 1 or 5, as appropriate, with MC diluent buffer. Liver and fecal samples were analyzed at a 1:10 dilution using MC sample diluent. Respiratory-swab and petri-dish samples were analyzed without further dilution. Dilutions were chosen to minimize false negatives, as this immunoassay was intended for screening purposes. Standards, controls, and sample extracts were run in duplicate. Absorbances were read at 450 nm using a microplate ELISA spectrophotometer. The minimum detection limit (MDL) varied by assay and sample type, but it ranged between 0.94 and 7.73 ng/g.

The selection criteria for confirmatory MMPB testing included those samples that (1) tested positive via ELISA and/or (2) had liver lesions potentially associated with MC toxicosis (see below). Confirmatory testing was performed at GreenWater Laboratories. The oxidation and analysis for total MCs and NODs was implemented as described in [23,44]. The MDL for MMPB was 10 ng/g. Confirmation of exposure via MMPB was required to link MC with liver lesions (see Section 2.4).

### 2.4. Liver Health Analysis

Liver samples were collected during the necropsy of code 2 and code 3 specimens using established protocols [40]. Formalin-fixed, paraffin-embedded, sectioned (3–7 µm), and H&E-stained samples were examined under light microscopy by board-certified veterinary pathologists experienced in wildlife health. Findings from gross and microscopic liver histopathology reports were reviewed for liver anomalies, including those that may be associated with MC exposure. These include hepatocellular necrosis, extramedullary hematopoiesis (EMH), primary liver cancer (i.e., hepatocellular carcinoma), lipidosis/vacuolation, fibrosis, hemorrhage, and/or inflammation (i.e., hepatitis) [23,45,46,47,48].

## 3. Results

### 3.1. Study Animals

**Figure 1:**
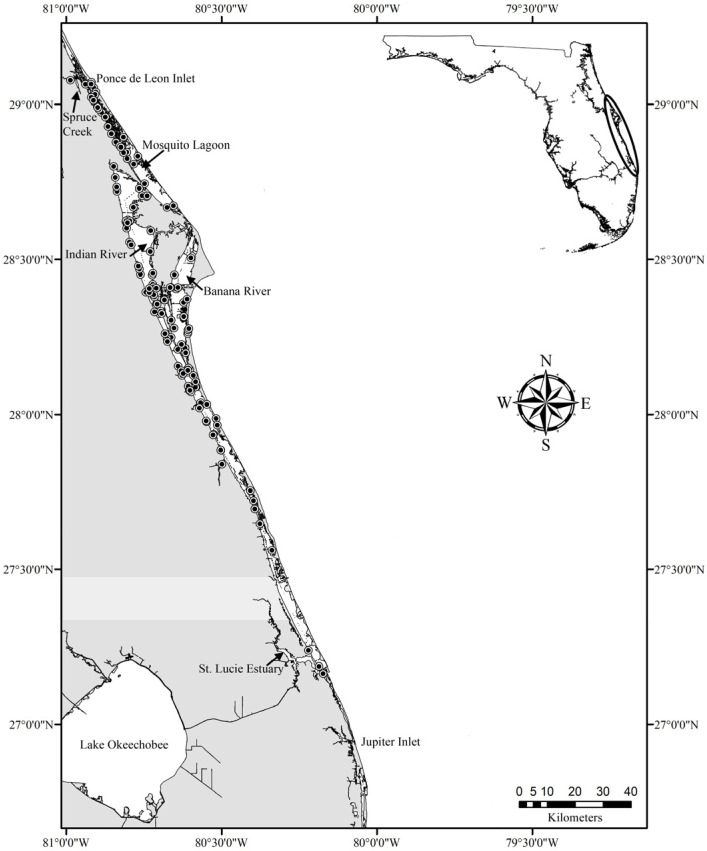
Common bottlenose dolphin (*Tursiops truncatus truncatus*) strandings (black dots) within the Indian River Lagoon (IRL) that were screened for microcystin (MC) exposure and liver lesions from 2005–2024. The study site extends along the east coast of Florida (inset map) between Ponce Inlet (Spruce Creek) and Jupiter Inlet.

A total of 156 bottlenose dolphins were evaluated (Table S1). Samples were collected between April 2005 and January 2024 (Table S1). Age class was known for 99% (*n* = 155) of cases, which comprised 41% adults (*n* = 63), 44% juveniles (*n* = 68), and 15% calves (*n* = 24) (Table S1). Of those with sex determined (98%, *n* = 153), 48% were female (*n* = 74), and 52% were male (*n* = 79) (Table S1). Stranding locations ranged from Port Orange, Volusia County (29.07765 N, −80.9865 W), in the north to Port Salerno, Martin County (27.1629 N, −80.173267 W) in the south with 94% (*n* = 147) of bottlenose dolphins from the northern IRL and 6% (*n* = 9) from the southern IRL (Figure 1). More than half of the strandings occurred in the rainy season (53%, *n* = 83) versus the dry season (47%, *n* = 73) (Table S1). The decomposition codes of the dolphins were as follows: code 1 = 0.5% (*n* = 1 case), code 2 = 45.5% (*n* = 71 cases), and code 3 = 54% (*n* = 84 cases).

### 3.2. Liver Health Analysis

**Figure 2:**
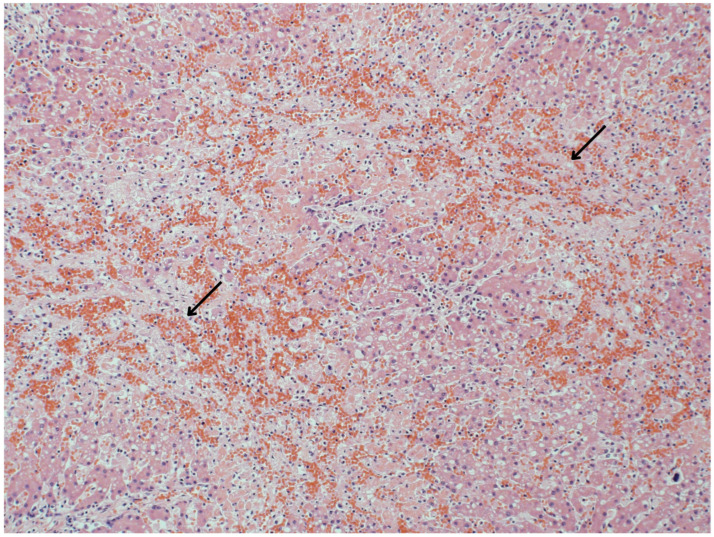
Histologic features of an IRL common bottlenose dolphin (Dolphin 11) with liver dysfunction (unknown etiology) as a cause of mortality. Acute hemorrhagic infarcts (arrows) and necrosis (marked, centrilobular, midzonal to submassive, and multifocal to coalescing) were observed microscopically (H&E; 300 dpi; 40×; scale bar = 40 µm). Image credit/histologic interpretation: Dr. David Rotstein.

**Figure 3:**
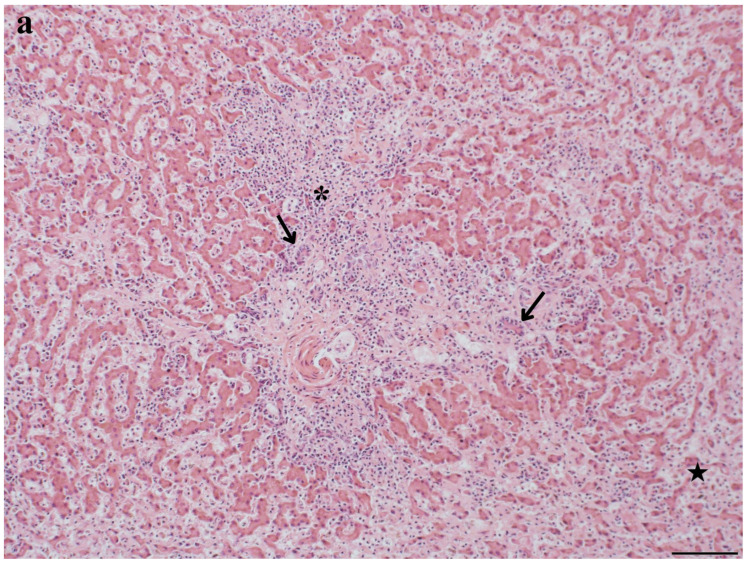

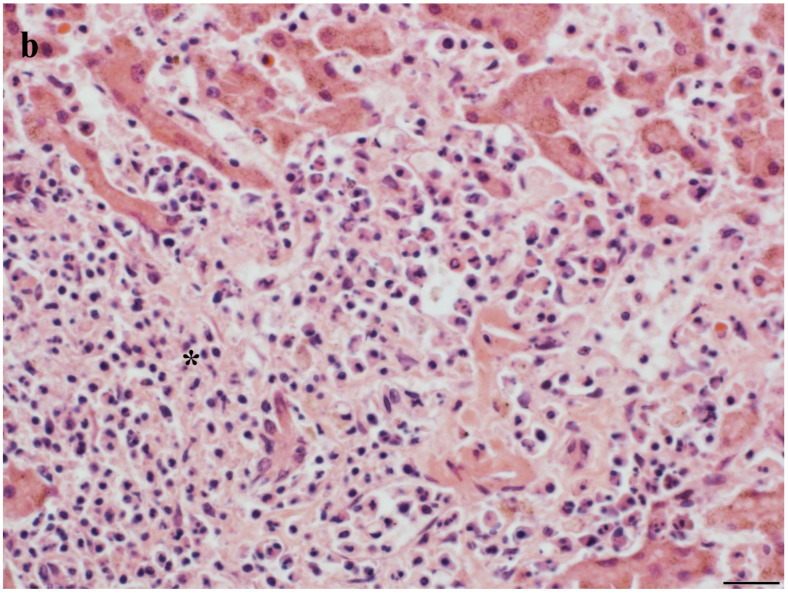
Histologic features of an IRL common bottlenose dolphin (Dolphin 12) with liver dysfunction (unknown etiology) as the cause of stranding. Periportal hepatitis (patchy and lymphoplasmacytic; asterisks), portal fibrosis (diffuse and moderate; star), and bile duct hyperplasia (arrows) were observed microscopically (H&E; 300 dpi with a scale bar). (**a**) 10×; scale bar = 100 µm (**b**) 40×; scale bar = 20 µm. Image credit/histologic interpretation: Dr. David Rotstein.

**Figure 4:**
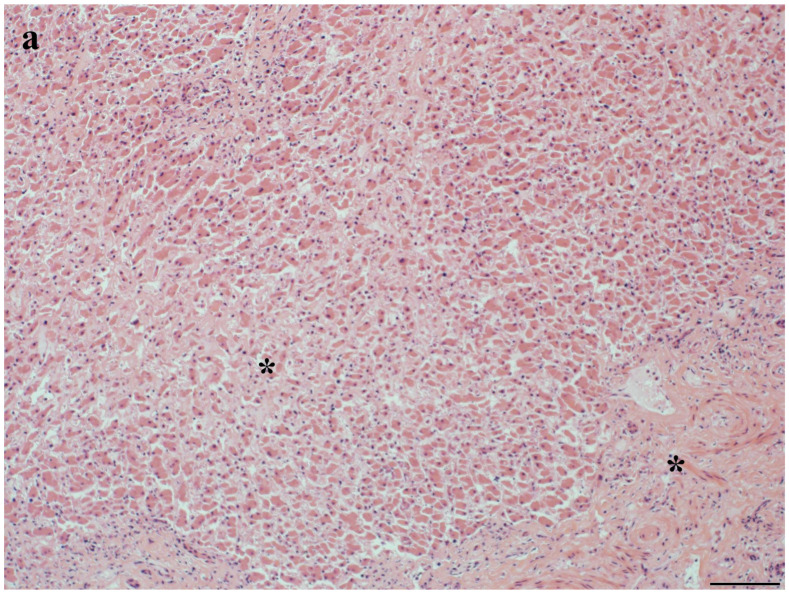

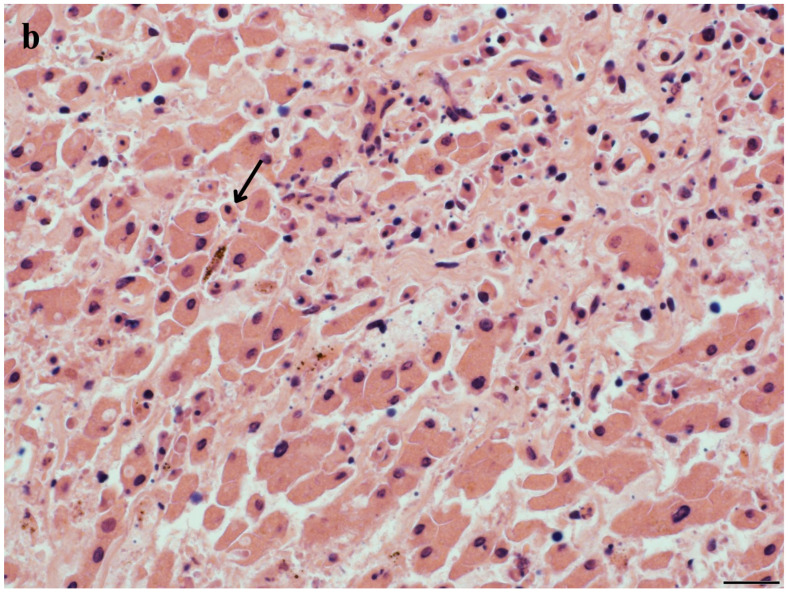
Histologic features of an IRL common bottlenose dolphin (Dolphin 13) with liver dysfunction (unknown etiology) as the cause of stranding and mortality. Portal hepatitis (mild, multifocal, chronic to active, lymphoplasmacytic, and neutrophilic), atrophy (mild and multifocal; arrow), and portal fibrosis (asterisks) were observed microscopically (H&E; 300 dpi with a scale bar). (**a**) 10×; scale bar = 100 µm (**b**) 40×; scale bar = 20 µm. Image credit/histologic interpretation: Dr. David Rotstein.

Liver histopathology was evaluated for 85% (*n* = 133) of bottlenose dolphins (Table S2). Of those, 50% (*n* = 66) had anomalies potentially associated with MC exposure, including inflammation (i.e., hepatitis), fibrosis, lipidosis/vacuolation, necrosis, EMH, and hemorrhage (Table 1). Other liver lesions included hyperplasia, hemosiderosis, congestion, atrophy, degeneration, infarct, vascular amyloidosis, active trematodes, and myeloid leukemia (Table 1). Lesions ranged from acute to chronic, mild to severe, and focal to diffuse (Table S2). Inflammation was characterized as lymphoplasmacytic (*n* = 16), neutrophilic (*n* = 6), histiocytic (*n* = 4), mononuclear (*n* = 1), fibrinous (*n* = 1), necrotic (*n* = 2), suppurative (*n* = 1), fibronecrotic (*n* = 1), necrosuppurative (*n* = 4), and/or granulomatous (*n* = 2) (Table S2). Primary hepatic dysfunction (unknown etiology) contributed to dolphin mortality in 3% (*n* = 4) of cases from the northern IRL (Table S2). Lesions included fibrosis, hyperplasia, hepatitis, necrosis, hemorrhage, and infarcts (Figure 2, Figure 3 and Figure 4). Liver dysfunction was not observed in bottlenose dolphins that stranded in the southern IRL. Liver anomalies were generally observed across age class, sex, and season, though EMH, congestion, and atrophy were not observed in calves, and infarct and degeneration were not observed in adults (Tables S1 and S2). The single cases of myeloid leukemia, active trematodes, and vascular amyloidosis were all observed in adult females (Tables S1 and S2).

### 3.3. Microcystin Analysis

For five bottlenose dolphin strandings, two or more sample types were available, leading to a total of 167 samples screened for MC via ELISA, including liver (*n* = 155), feces (*n* = 6), respiratory swabs (*n* = 4), and respiratory vapor petri dishes (*n* = 2) (Table S2). Microcystin was detected via ELISA in 6% (*n* = 10) of samples, including liver (*n* = 8) and feces (*n* = 2) (Table 2). Microcystin was not detected in the liver samples of the two individuals (Dolphins 9 and 10) with MC detected in feces. All ELISA-positive samples were obtained from dolphins that stranded in the northern IRL. Sixty percent (*n* = 6) of ELISA-positive samples were from strandings that occurred during the rainy season, while 40% (*n* = 4) were from strandings that occurred during the dry season (Tables S1 and S2). The two highest ELISA MC levels (34.2 ng/g) were from stranding events that occurred during the dry season (Tables S1 and S2). Over the two-decade long study period, MC was detected via ELISA in 35% (*n* = 7) of years. During each of the following years, 10% (*n* = 1/per annum) of dolphins with an ELISA-positive sample stranded: 2005, 2007, 2012, and 2015, while 20% (*n* = 2/per annum) stranded in 2008, 2013, and 2023 (Tables S1 and S2). Fifty percent of ELISA-positive samples were from females (*n* = 5), and 50% were from males (*n* = 5), while the age class included 10% (*n* = 1) adults, 50% (*n* = 5) juveniles, and 40% (*n* = 4) calves (Tables S1 and S2). Liver histopathology was available for 60% (*n* = 6) of these individuals. A liver lesion (lipidosis) was noted in 33% (*n* = 2) of ELISA-positive cases, with liver dysfunction contributing to dolphin mortality in one case (Dolphin 5) (Table 2). However, MMPB testing (*n* = 56) did not confirm MC in any specimen (Table S2).

### 3.4. Aerosol Microcystin Screening

Spike/recovery tests of respiratory-vapor petri-dish samples yielded recovery values ranging from 57.5 to 140% for concentrations between 0.15 and 1 ng MC/mL extract, while 0% recovery was observed for concentrations between 0.03 and 0.2 ng MC/mL extract (Table S3). The sample blank demonstrated false positive recovery (0.22 ng MC/mL extract). Spike/recovery tests for respiratory-swab samples yielded recovery values ranging from 120 to 833% for concentrations between 0.03 and 1 ng MC/mL extract (Table S4). The recovery value for 1 ng MC/mL extract (120%) was at the upper end of the acceptable range (80–120%) [49]. After the false positive recovery observed in both blank and standard 0 spiked swabs (0.18 ng MC/mL extract) (Table S4) was accounted for, spiked concentrations between 0.2 and 1 ng MC/mL extract demonstrated adequate MC recovery (87.5–102%) [49].

Microcystin was not detected in respiratory-vapor petri-dish samples from two live-stranded IRL bottlenose dolphins (Table S2). While MC was also not detected in the liver or feces of one individual with a petri-dish sample, other sample types were not available for toxin screening in the other case. Microcystin-like activity was detected in the four respiratory-swab samples. Microcystin was not detected in the liver samples of these individuals, though a positive fecal sample was observed in one case (Dolphin 9, see Section 2.2).

## 4. Discussion

This study represents the first survey for MC in IRL bottlenose dolphins. The detection rate via ELISA in bottlenose dolphins (6%) was similar to green sea turtles (5–7.5%), but lower than bull sharks (20%) in the IRL [5,10]. However, MC exposure was not confirmed via MMPB. Further investigation is required to confirm exposure and its relationship with liver anomalies in IRL bottlenose dolphins. The inability to confirm positive ELISA results may be due to false positives or differences in test detection limits or toxin forms recognized (i.e., free, bound, degraded, and conjugated) [23,32]. For example, MC levels (3.3–6.8 ng/g) in five liver samples were below the MDL of MMPB (10 ng/g). These samples may require another method for confirmation (e.g., ICP-MS), or they may represent false positives.

Statistical analysis was not performed to evaluate the impacts of sex, age class, season, year, or stranding location on MC status due to the limited number of ELISA-positive samples and lack of MMPB confirmation. However, most of the ELISA-positive samples in this study were obtained from immature dolphins or stranding events that occurred during the rainy season and in the northern IRL, and no sex-based patterns were observed. Age-related differences in MC bioaccumulation may be due to differential prey selection or MC absorption, metabolism, storage, or excretion [50,51]. Though most ELISA-positive samples were obtained from bottlenose dolphins that stranded during the rainy season, the highest liver values were from those that stranded during the dry season. The highest MC water levels were measured in the IRL during the rainy season due to contaminated Lake Okeechobee releases [26,27,28,52]. However, MC was previously detected in IRL wildlife tissue samples across seasons, even when no known blooms were occurring [5,10,25]. The persistence of MC in the water column, as well as the hydrophobic nature of some congeners, may result in MC bioaccumulation in IRL wildlife tissues, regardless of bloom status or season [10,24]. Microcystin was previously detected in IRL water samples in 2005, 2013, and 2023, corresponding to MC-producing blooms in the southern IRL [26,27,53]. It is possible that these blooms were also responsible for MC exposure to IRL bottlenose dolphins during those time periods. The bottlenose dolphin that stranded in 2015 and tested MC-positive via ELISA may have been exposed to a similar toxin source as green sea turtles during that year [10]. However, since green sea turtles are herbivores and feed at a different trophic level than bottlenose dolphins, they were unlikely to consume the same MC vector. This study is the first report of MC detection within the lagoon in 2007, 2008, and 2012, as well as the first report of MC exposure to IRL wildlife in 2023. Notably, the ELISA-positive dolphins that stranded in 2007 (Dolphin 2) and 2008 (Dolphin 4) also tested positive for PbTx and STX [54], suggesting that environmental conditions may have supported HAB toxin production. Other HAB events occurred in the northern IRL in 2012, though primarily after the ELISA-positive dolphin (Dolphin 5) stranded that year [55].

The geographic distribution and foraging habits of IRL bottlenose dolphins may provide insight into MC exposure risk. Bottlenose dolphins in the lagoon exhibit site fidelity and can be divided into discrete communities based on habitat use and social interactions [33,56,57]. Though MC has been detected in water and wildlife tissue samples from throughout the IRL [5,10,25], the toxin is present at higher concentrations in the southern portion and was not detected in river otters found as road victims primarily in the northern portion [25,58]. Since most bottlenose dolphins in this study were also observed to have stranded in the northern portion of the IRL, the subpopulations at risk for higher-level exposure may not be adequately represented herein. Most of the ELISA-positive samples, as well as the highest ELISA MC levels measured, were obtained from stranding events in the north-central IRL and Banana River. At present, a source of MC to this region of the lagoon is unknown. However, the area coincides with a distinct social community of bottlenose dolphins [57], and it may be impacted by urban/residential, agricultural, and industrial inputs [55]. Moreover, MC has been detected in nearby freshwater bodies that may have inputs into the north-central IRL [59]. To the authors’ knowledge, there are no reports describing MC loads in IRL bottlenose dolphin prey. The primary prey of bottlenose dolphins in the lagoon includes spotted sea trout (*Cynoscion nebulosus*), silver perch (*Bairdiella chrysoura*), Atlantic croaker (*Micropogonias undulates*), oyster toadfish (*Opsanus tau*), and striped mullet (*Mugil cephalus*) [34,60]. Striped mullet were hypothesized as a MC vector based on their dominance in the gastric contents of IRL bull sharks with confirmed exposure [5].

Microcystin exposure doses, and subsequent tissue levels and lesions, that correspond to acute and chronic disease in cetaceans are unknown. Total hepatic levels as high as 14.3 ± 5.6 ng/g were not associated with overt toxicosis in estuarine bottlenose dolphins in northeast Florida [32]. Similarly, liver concentrations as high as 82.5 ng/g and 100.2 ng/g were not associated with overt MC toxicosis in IRL bull sharks or green sea turtles, respectively [5,10]. Liver concentrations ranging from 1.36 to 387 ng/g were measured in southern sea otters (*Enhydra lutris nereis*) with MC-induced acute liver failure, although MC was not detected in every case, and the time lapse since exposure was unknown [45]. Further research may help elucidate background versus acute and chronic MC toxicosis levels and associated lesions in IRL megafauna.

Numerous liver lesions were observed in IRL bottlenose dolphins in this study. Since MC was not linked with these anomalies, other causes (e.g., current or previous metazoal, protozoal, fungal, bacterial, or viral infection, other HAB biotoxins, heavy metals, and metabolic/nutritional disease) should be considered [61,62,63]. Further diagnostic tests, including special stains of histopathology slides (e.g., Masson’s trichrome), infectious disease serology and culture, and heavy metal screening, may help elucidate a definitive diagnosis. Microcystin-induced liver lesions have not been reported in cetaceans, including bottlenose dolphins with confirmed exposure in northeast Florida [32]. Hepatic fibrosis of unknown etiology, as well as hepatic lipidosis, are common in free-ranging bottlenose dolphins and were previously observed at similar prevalences (~30% and ≥12%, respectively) [61,62,64]. Mercury bioaccumulation in bottlenose dolphins has been associated with hepatic necrosis, hepatitis, and hepatic lipidosis in western Florida [65], as well as liver enzyme elevations in the IRL [66,67], and they may have contributed to the liver lesions observed herein. Though liver neoplasia is uncommon in bottlenose dolphins, adenoma, reticuloendotheliosis, and immunoblastic malignant lymphoma have been reported [63,68,69,70], the latter of which was associated with elevated concentrations of polychlorinated biphenyl congeners [63]. Myeloid leukemia (unknown tissue) was also previously reported in a bottlenose dolphin that stranded along the Gulf Coast of Texas [71]. Chronic lymphocytic, plasmocytic, and/or neutrophilic hepatitis was previously reported in captive bottlenose dolphins and may be associated with metabolic disease [61]. Liver lesions in IRL bottlenose dolphins were present across age class, sex, and season. No age or sex predilections for hepatic hemosiderosis or lipidosis were also observed in bottlenose dolphins under human-managed care, though hepatitis was more common in older individuals [61]. The presence of underlying liver disease may increase the vulnerability of IRL bottlenose dolphins to HAB toxin exposure [72], especially since this stock is immunocompromised, has experienced several unusual mortality events (UMEs), and is subjected to numerous other natural and anthropogenic stressors [9,18,33,73,74].

Microcystin was not detected in respiratory-vapor petri-dish samples of bottlenose dolphins in this study. Microcystin-like activity detected in respiratory swabs of bottlenose dolphins were considered false positives as these samples did not likely undergo sufficient extraction (i.e., SPE cleaning) since debris was visible [23]. These methods were evaluated to determine whether aerosol droplets may serve as a pre-mortem tool for MC screening of free-ranging populations. Generally, toxin-spiked petri dishes and swabs yielded inadequate recovery of standards, while the blank samples demonstrated false positive recovery. However, the highest toxin-spiked swab (1 ng MC/mL extract) yielded adequate MC recovery [49]. Spiked MC concentrations were considered ecologically relevant, as they aligned with the levels detected via nasal swabs in human exposure studies [29,43]. Notably, a similar false positive recovery level (0.2 ng) was observed in a human MC nasal swab study [43]. False positive ELISA results may have occurred if sample extraction was not sufficient [23]. Further validation of sterile cotton-tip swabs, as well as other materials (e.g., a Nitex membrane-covered petri dish), for the collection, preservation, and detection of MC from aerosol droplets is recommended [75]. Further research is also required to determine MC aerosol levels that correspond to background exposure versus acute and chronic MC toxicosis in free-ranging cetaceans, although concentrations up to 5 ng were not associated with obvious liver disease in humans [43].

## 5. Conclusions

Microcystin was detected via ELISA but not confirmed through MMPB, in bottlenose dolphins in the IRL estuary system. Thus, while the population experienced liver insult, these lesions were not linked with MC exposure. More sensitive tests (e.g., ICP-MS) may be required to confirm MC exposure in this population, or ELISA-positive samples may represent false positives. Other causes (e.g., infectious agents, metabolic/nutritional disease, and/or other environmental contaminants) should be considered for the liver lesions observed. Future research is recommended to characterize acute versus chronic versus background MC exposure in bottlenose dolphins. Investigation of MC loads in IRL bottlenose dolphin prey species may better elucidate the risk and pathway of toxin exposure to this immunocompromised stock [74]. Continued MC screening is recommended in bottlenose dolphins with suggestive liver lesions, hepatic dysfunction, or found within the southern IRL, SLE, or during a bloom event.

## Figures and Tables

**Table 1:** Types and incidence of histologic liver anomalies observed in IRL common bottlenose dolphins (2005–2023). Lesions potentially associated with MC exposure are shown in italics.

Liver Lesion	Incidence in IRL Bottlenose Dolphins
*Hepatitis*	24% (*n* = 32)
*Fibrosis*	23% (*n* = 31)
*Lipidosis/Vacuolation*	11% (*n* = 15)
*Necrosis*	11% (*n* = 14)
*Extramedullary hematopoiesis*	5% (*n* = 6)
*Hemorrhage*	2% (*n* = 3)
Hyperplasia	9% (*n* = 12)
Hemosiderosis	6% (*n* = 8)
Congestion	5% (*n* = 6)
Atrophy	3% (*n* = 4)
Degeneration	2% (*n* = 3)
Infarct	2% (*n* = 2)
Vascular amyloidosis	1% (*n* = 1)
Trematodes	1% (*n* = 1)
Myeloid leukemia	1% (*n* = 1)

**Table 2:** Indian River Lagoon common bottlenose dolphins that were positive for MC via ADDA-ELISA (2005–2024), the sample type analyzed, concentration detected (ng/g) via ELISA and the 2-methyl-3-methoxy-4-phenylbutyric acid technique (MMPB), and liver lesions present (NSF = no significant findings, NA = not available for histology or MMPB testing, and MDL = minimum detection limit).

Animal ID	Sample Type	ELISA MC Level (ng/g)	MMPB MC Level (ng/g)	Liver Lesions
Dolphin 1	Liver	6.7	<MDL	NSF
Dolphin 2	Liver	6.8	<MDL	NA
Dolphin 3	Liver	34.2	<MDL	NA
Dolphin 4	Liver	4.4	<MDL	NSF
Dolphin 5	Liver	34.2	<MDL	Lipidosis, Dysfunction
Dolphin 6	Liver	3.3	<MDL	NSF
Dolphin 7	Liver	26.2	<MDL	Lipidosis
Dolphin 8	Liver	6.8	<MDL	NA
Dolphin 9	Feces Liver	2.3 <MDL	NA NA	NSF
Dolphin 10	Feces Liver	3.0 <MDL	NA NA	NA

## Data Availability

The original contributions presented in the study are included in the article/supplementary material; further inquiries can be directed to the corresponding author.

## Supplementary Materials

The following supporting information can be downloaded at: https://www.mdpi.com/article/10.3390/toxics12120858/s1, Table S1. Raw data for Indian River Lagoon (IRL) common bottlenose dolphins (*Tursiops truncatus truncatus*) (*n* = 156) evaluated for microcystin (MC) exposure and liver lesions (2005–2024), including stranding location, date, and season, and demographic information (TL = Total Length, NA = data not available). Table S2. Raw data for IRL common bottlenose dolphins (*n* = 156) evaluated for MC exposure (2005–2024), including sample type tested, MC values, and liver histopathology results (NA = data or samples not available, MDL = minimum detection limit, MMPB = 2-methyl-3-methoxy-4-phenylbutyric acid technique, NSF = no significant histopathological findings). Table S3. The actual MC level versus the recovered concentration via ADDA-ELISA of spiked sterile respiratory vapor petri dish samples. Respiratory vapor is under evaluation as a non-invasive MC detection method in free-ranging bottlenose dolphins. Table S4. The actual MC level versus the recovered concentration via ADDA-ELISA of spiked sterile cotton tip swabs. Swabs of respiratory fluid/vapor are under evaluation as a non-invasive MC detection method in free-ranging bottlenose dolphins.
