# Supplementary material for: Liver Lesions in Estuarine Dolphins in the Indian River Lagoon, Florida: Does Microcystin Play a Role?
Source: Toxics. 2024 Nov 27;12(12):858. doi: 10.3390/toxics12120858 (PMC11678953; doi:10.3390/toxics12120858)
Supplement: Supplementary file 1 [file toxics-12-00858-s001.zip › toxics-3241060-supplementary.pdf]

Table S1. Raw data for Indian River Lagoon (IRL) common bottlenose dolphins (*Tursiops truncatus truncatus)* (*n* = 156) evaluated for microcystin (MC) exposure and liver lesions (2005-2024), including stranding location, date, and season, and demographic information (TL = Total Length, NA = data not available).

Sender ID	Publication ID	TL (cm)	Sex	Age class	Latitude	Longitude	Season	Stranding date	Decomp code
HBOI-1303-Tt	NA	214	Male	Juvenile	27.72123	-80.397	Dry	2/18/2013	2
HBOI-1309-Tt	NA	201	Male	Juvenile	27.69469	-80.3931	Rainy	7/13/2013	3
HBOI-1409-Tt	NA	275	Female	Adult	27.69449	-80.39328	Rainy	8/8/2014	3
HBOI-1601-Tt	NA	290.5	Male	Adult	27.18663	-80.18649	Dry	2/17/2016	2
HBOI-1602-Tt	NA	214	Female	Juvenile	27.56107	-80.33757	Dry	2/27/2016	2
HBOI-2101-Tt	NA	188	Female	Juvenile	27.1629	-80.17327	Dry	3/12/2021	3
HBOI-2201-Tt	NA	256	Male	Adult	27.6463	-80.37629	Dry	2/4/2022	2
HBOI-2203-Tt	NA	272.1	Male	Adult	27.23801	-80.22057	Dry	3/27/2022	2
HBOI-2205-Tt	NA	256	Male	Adult	27.75385	-80.40728	Dry	4/13/2022	2
Hubbs-0510-Tt	NA	109	Female	Calf	28.70565	-80.75585	Dry	4/9/2005	2
Hubbs-0517-Tt	*Dolphin 1*	259	Male	Adult	28.02033	-80.57143	Rainy	6/14/2005	3
Hubbs-0523-Tt	NA	175	Female	Juvenile	29.06528	-80.91943	Rainy	7/20/2005	2
Hubbs-0541-Tt	NA	245.3	Male	Juvenile	28.14475	-80.60187	Dry	12/26/2005	2
Hubbs-0630-Tt	NA	236	Female	Adult	28.73278	-80.8374	Rainy	5/24/2006	2
Hubbs-0701-Tt	NA	249	Male	Adult	28.14922	-80.60703	Dry	1/1/2007	3
Hubbs-0709-Tt	*Dolphin 2*	222	Male	Juvenile	28.09044	-80.605	Dry	1/18/2007	2

Hubbs-0717-Tt	NA	250	Male	Adult	28.20893	-80.6407	Dry	2/11/2007	2
Hubbs-0723-Tt	NA	205	Male	Juvenile	28.82517	-80.80385	Dry	3/12/2007	3
Hubbs-0760-Tt	NA	277	Male	Adult	28.87728	-80.83985	Rainy	9/5/2007	3
Hubbs-0805-Tt	NA	231	Female	Adult	28.14067	-80.62993	Dry	1/30/2008	2
Hubbs-0808-Tt	NA	236	Female	Adult	28.1556	-80.63967	Dry	2/4/2008	3
Hubbs-0825-Tt	NA	192	Female	Juvenile	28.2251	-80.6291	Dry	4/19/2008	3
Hubbs-0827-Tt	*Dolphin 3*	226	Female	Juvenile	28.24343	-80.67542	Dry	4/25/2008	3
Hubbs-0828-Tt	NA	115	Male	Calf	28.39407	-80.74361	Rainy	5/2/2008	2
Hubbs-0831-Tt	*Dolphin 4*	207	Male	Juvenile	28.40893	-80.64032	Rainy	5/12/2008	3
Hubbs-0837-Tt	NA	241	Male	Juvenile	28.62247	-80.79531	Rainy	5/31/2008	3
Hubbs-0838-Tt	NA	105	Female	Calf	28.24799	-80.66184	Rainy	6/2/2008	2
Hubbs-0848-Tt	NA	243	Female	Adult	28.59987	-80.80517	Rainy	6/26/2008	3
Hubbs-0854-Tt	NA	240	Female	Adult	28.8442	-80.815	Rainy	7/7/2008	3
Hubbs-0875-Tt	NA	265	Male	Adult	28.61426	-80.8041	Rainy	8/18/2008	2
Hubbs-0901-Tt	NA	163	Male	Juvenile	28.38566	-80.71803	Dry	1/9/2009	2
Hubbs-0910-Tt	NA	192	Male	Juvenile	28.5561	-80.7962	Dry	3/6/2009	2
Hubbs-0915-Tt	NA	240	Female	Adult	28.61878	80.8036	Dry	3/22/2009	2
Hubbs-0924-Tt	NA	249	Female	Adult	28.70242	-80.74132	Rainy	5/6/2009	2
Hubbs-0940-Tt	NA	178	Female	Juvenile	28.14595	-80.60407	Rainy	8/28/2009	2

Hubbs-0942-Tt	NA	181	Male	Juvenile	28.62119	-80.80757	Rainy	9/10/2009	3
Hubbs-0955-Tt	NA	241	Female	Adult	28.36085	-80.62213	Dry	12/23/2009	3
Hubbs-1045-Tt	NA	198	Male	Juvenile	28.03688	-80.56754	Dry	4/22/2010	3
Hubbs-1071-Tt	NA	198	Male	Juvenile	28.08723	-80.58383	Rainy	10/19/2010	3
Hubbs-1073-Tt	NA	252	Male	Adult	28.56892	80.78058	Rainy	11/4/2010	2
Hubbs-1107-Tt	NA	164	Female	Juvenile	28.95906	-80.8736861	Dry	2/7/2011	3
Hubbs-1122-Tt	NA	255	Male	Adult	28.70483	-80.73913	Rainy	6/22/2011	2
Hubbs-1132-Tt	NA	217	Male	Juvenile	28.10481	-80.583465	Rainy	7/22/2011	2
Hubbs-1202-Tt	NA	210	Female	Juvenile	28.45467	-80.72085	Dry	1/8/2012	3
Hubbs-1212-Tt	NA	212	Male	Juvenile	27.88414	-80.50269	Dry	2/28/2012	2
Hubbs-1216-Tt	NA	205	Male	Juvenile	27.96481	-80.5136	Dry	3/8/2012	2
Hubbs-1217-Tt	*Dolphin 5*	161	Male	Juvenile	28.25988	-80.68222	Dry	3/16/2012	2
Hubbs-1227-Tt	NA	278	Male	Adult	28.08682	-80.60546	Rainy	5/1/2012	3
Hubbs-1235-Tt	NA	249	Female	Adult	28.12551	-80.5907972	Rainy	6/4/2012	2
Hubbs-1249-Tt	NA	203	Male	Juvenile	28.13742	80.60134	Rainy	8/20/2012	2
Hubbs-1255-Tt	NA	122	Male	Calf	28.03297	80.59014	Rainy	9/3/2012	3
Hubbs-1307-Tt	NA	182	Female	Juvenile	28.72823	-80.7646861	Dry	2/5/2013	2
Hubbs-13102-Tt	*Dolphin 6*	117	Male	Calf	28.39273	-80.73218	Rainy	10/16/2013	3
Hubbs-13104-Tt	NA	199	Female	Juvenile	28.79985	-80.84718	Rainy	11/7/2013	3

Hubbs-13105-Tt	NA	255	Female	Adult	28.66742	-80.67497	Rainy	11/7/2013	3
Hubbs-13107-Tt	NA	225	NA	Juvenile	27.8841	-80.50244	Rainy	11/12/2013	3
Hubbs-1310-Tt	NA	245	Female	Adult	28.27844	-80.65277	Dry	2/21/2013	2
Hubbs-1312-Tt	NA	256	Female	Adult	28.4095	-80.66309	Dry	2/24/2013	3
Hubbs-1315-Tt	NA	231	Female	Adult	28.47799	-80.767881	Dry	3/8/2013	2
Hubbs-1319-Tt	NA	243	Male	Juvenile	28.89387	-80.8154027	Dry	3/11/2013	2
Hubbs-1328-Tt	NA	233	Male	Juvenile	28.86653	-80.82766	Dry	3/23/2013	3
Hubbs-1331-Tt	NA	245	Male	Juvenile	28.59205	-80.72756	Dry	4/1/2013	3
Hubbs-1332-Tt	NA	242	Female	Adult	28.30365	-80.662231	Dry	4/2/2013	2
Hubbs-1333-Tt	NA	208	NA	Juvenile	28.03181	-80.54741	Dry	4/3/2013	3
Hubbs-1334-Tt	NA	243	Female	Adult	28.66765	-80.78345	Dry	4/3/2013	3
Hubbs-1351-Tt	NA	255	Male	Adult	28.13685	80.60194	Rainy	5/11/2013	3
Hubbs-1352-Tt	NA	243	Male	Juvenile	28.13147	-80.62346	Rainy	5/17/2013	3
Hubbs-1353-Tt	*Dolphin 7*	114	Female	Calf	28.40909	-80.66553	Rainy	5/17/2013	2
Hubbs-1355-Tt	NA	233	Female	Adult	28.44987	-80.65163	Rainy	5/22/2013	3
Hubbs-1371-Tt	NA	257	Female	Adult	28.08316	-80.59538	Rainy	6/23/2013	3
Hubbs-1383-Tt	NA	224	Female	Juvenile	28.86227	-80.82077	Rainy	8/7/2013	2
Hubbs-1387-Tt	NA	247	Male	Adult	28.6767	80.64454	Rainy	8/16/2013	3
Hubbs-1408-Tt	NA	160	Male	Calf	28.32422	-80.69983	Dry	1/21/2014	3

Hubbs-1416-Tt	NA	251	Female	Adult	28.84632	-80.80469	Dry	2/13/2014	3
Hubbs-1431-Tt	NA	229	Female	Juvenile	28.46823	-80.76432	Rainy	5/2/2014	2
Hubbs-1433-Tt	NA	181	Male	Juvenile	28.12547	-80.62738	Rainy	5/28/2014	3
Hubbs-1440-Tt	NA	126	Male	Calf	29.02275	-80.91926	Rainy	7/18/2014	3
Hubbs-1448-Tt	NA	258	Male	Adult	29.05343	-80.910898	Rainy	7/30/2014	2
Hubbs-1450-Tt	NA	252	Male	Adult	28.99007	-80.89874	Rainy	7/6/2014	3
Hubbs-1451-Tt	NA	208	Male	Juvenile	29.02261	-80.91727	Rainy	8/8/2014	2
Hubbs-1466-Tt	NA	175	Male	Juvenile	29.06463	-80.9379	Rainy	8/27/2014	2
Hubbs-1468-Tt	NA	135	Female	Calf	28.99307	-80.89988	Rainy	9/2/2014	2
Hubbs-1486-Tt	NA	108	Male	Calf	28.213	-80.6201	Rainy	10/18/2014	2
Hubbs-1491-Tt	NA	252	Male	Adult	29.03981	-80.90428	Rainy	11/2/2014	2
Hubbs-1496-Tt	NA	120	Female	Calf	28.40886	-80.64023	Dry	12/3/2014	3
Hubbs-1501-Tt	NA	250	Female	Adult	28.40432	-80.64549	Dry	1/22/2015	3
Hubbs-1506-Tt	NA	255	Male	Adult	28.74399	-80.74798	Dry	3/3/2015	2
Hubbs-1508-Tt	*Dolphin 8*	172	Female	Juvenile	28.92848	-80.86535	Dry	3/20/2015	3
Hubbs-1509-Tt	NA	242	Female	Adult	28.54705	-80.79073	Dry	3/20/2015	3
Hubbs-1520-Tt	NA	240	NA	NA	28.62748	-80.80167	Rainy	6/13/2015	2
Hubbs-1523-Tt	NA	194	Female	Juvenile	28.3148	-80.62161	Rainy	6/29/2015	3
Hubbs-1525-Tt	NA	173	Male	Juvenile	28.52539	-80.72951	Rainy	7/1/2015	2

Hubbs-1544-Tt	NA	109	Female	Calf	28.76397	-80.84208	Rainy	9/11/2015	2
Hubbs-1549-Tt	NA	160	Male	Calf	29.01305	-80.91123	Rainy	10/13/2015	3
Hubbs-1603-Tt	NA	231	Female	Adult	28.08199	-80.60253	Dry	2/3/2016	3
Hubbs-1607-Tt	NA	204	Male	Juvenile	29.00842	-80.90925	Dry	2/27/2016	3
Hubbs-1617-Tt	NA	115	Female	Calf	28.40708	-80.70925	Rainy	7/2/2016	2
Hubbs-1656-Tt	NA	254	Female	Adult	29.04301	-80.90957	Rainy	10/26/2016	2
Hubbs-1659-Tt	NA	127	Female	Calf	28.71945	-80.83612	Rainy	11/4/2016	3
Hubbs-1666-Tt	NA	250	Male	Adult	28.90501	-80.85441	Dry	2/14/2016	2
Hubbs-1728-Tt	NA	244	Female	Adult	28.35707	-80.70879	Rainy	7/26/2017	2
Hubbs-1730-Tt	NA	190	Male	Juvenile	28.98446	-80.89449	Rainy	8/26/2017	3
Hubbs-1743-Tt	NA	157.5	Female	Calf	29.07167	-80.91859	Dry	12/26/2017	2
Hubbs-1801-Tt	NA	208	Male	Juvenile	28.45116	-80.76106	Dry	1/9/2018	3
Hubbs-1802-Tt	NA	218	Male	Juvenile	28.80698	-80.78237	Dry	1/17/2018	2
Hubbs-1804-Tt	NA	269	Male	Adult	28.97401	-80.8857	Dry	3/7/2018	2
Hubbs-1805-Tt	NA	252	Female	Adult	28.37862	-80.71583	Dry	4/4/2018	2
Hubbs-1812-Tt	NA	257	Female	Adult	27.83887	-80.49902	Rainy	6/1/2018	3
Hubbs-1815-Tt	NA	234	Female	Adult	29.03164	-80.90726	Rainy	6/29/2018	3
Hubbs-1830-Tt	NA	242	Male	Juvenile	28.61776	-80.80237	Rainy	8/11/2018	2
Hubbs-1840-Tt	NA	120	Male	Calf	28.36872	-80.68495	Rainy	10/7/2018	2

Hubbs-1844-Tt	NA	224	Female	Juvenile	29.01285	-80.91218	Rainy	11/5/2018	3
Hubbs-1848-Tt	NA	229	Female	Juvenile	28.33101	-80.69452	Dry	12/12/2018	3
Hubbs-1903-Tt	NA	279	Male	Adult	28.27069	-80.60804	Dry	1/30/2019	3
Hubbs-1904-Tt	NA	240	Female	Adult	27.93537	-80.52574	Dry	2/1/2019	2
Hubbs-1911-Tt	NA	251	Female	Adult	28.08783	-80.58149	Dry	4/6/2019	2
Hubbs-1914-Tt	NA	191	Male	Juvenile	28.08594	-80.58825	Rainy	6/2/2019	3
Hubbs-1919-Tt	NA	263	Male	Adult	28.4052	-80.6478	Rainy	7/20/2019	3
Hubbs-1934-Tt	NA	223	Female	Juvenile	28.86123	-80.82343	Rainy	10/12/2019	3
Hubbs-2003-Tt	NA	226	Male	Juvenile	28.1502	-80.6067	Dry	1/31/2020	3
Hubbs-2004-Tt	NA	235	Female	Adult	28.09041	-80.6071	Dry	2/9/2020	3
Hubbs-2018-Tt	NA	218	Male	Juvenile	28.62393	-80.7954	Rainy	6/1/2020	3
Hubbs-2025-Tt	NA	236	Female	Adult	28.3713	-80.61078	Rainy	7/26/2020	3
Hubbs-2046-Tt	NA	246	Male	Adult	28.67307	-80.65445	Dry	12/12/2020	3
Hubbs-2048-Tt	NA	210	Female	Juvenile	28.26002	-80.60898	Dry	12/18/2020	3
Hubbs-2102-Tt	NA	205	Female	Juvenile	28.1418	-80.6085	Dry	1/11/2021	3
Hubbs-2104-Tt	NA	203	Male	Juvenile	28.26614	-80.60791	Dry	1/11/2021	3
Hubbs-2105-Tt	NA	198	Male	Juvenile	28.27279	-80.60802	Dry	1/30/2021	3
Hubbs-2115-Tt	NA	235	Male	Juvenile	28.07677	-80.60389	Dry	2/3/2021	3
Hubbs-2117-Tt	NA	198	Female	Juvenile	29.07765	-80.9865	Dry	3/15/2021	1

Hubbs-2121-Tt	NA	132	Male	Calf	28.13572	-80.60907	Dry	4/9/2021	3
Hubbs-2127-Tt	NA	122	Male	Calf	28.62408	-80.80693	Rainy	5/18/2021	3
Hubbs-2128-Tt	NA	205	Male	Juvenile	28.44906	-80.72326	Rainy	6/27/2021	3
Hubbs-2129-Tt	NA	201	Male	Juvenile	28.40602	-80.73235	Rainy	6/26/2021	2
Hubbs-2133-Tt	NA	211	Female	Juvenile	28.07156	-80.59812	Rainy	6/29/2021	2
Hubbs-2146-Tt	NA	242	Female	Adult	27.97856	-80.54969	Rainy	7/30/2021	3
Hubbs-2148-Tt	NA	119	Male	Calf	28.35504	-80.7071	Rainy	11/7/2021	2
Hubbs-2206-Tt	NA	222	Female	Juvenile	27.93276	-80.52757	Rainy	11/12/2022	3
Hubbs-2207-Tt	NA	235	Female	Adult	28.32979	-80.71554	Dry	3/4/2022	2
Hubbs-2211-Tt	NA	231	Female	Adult	27.98672	-80.5173	Dry	4/19/2022	2
Hubbs-2214-Tt	NA	225	Female	Juvenile	27.97588	-80.54814	Rainy	5/12/2022	2
Hubbs-2216-Tt	NA	194	Male	Juvenile	28.1978	-80.61527	Dry	3/7/2022	3
Hubbs-2224-Tt	NA	265	Male	Adult	28.21359	-80.61744	Rainy	6/5/2022	3
Hubbs-2225-Tt	NA	195	Male	Juvenile	28.41974	-80.72172	Rainy	7/22/2022	3
Hubbs-2236-Tt	NA	275	Male	Adult	28.41088	-80.71996	Rainy	8/28/2022	3
Hubbs-2238-Tt	NA	114	Female	Calf	28.83306	-80.76937	Rainy	7/21/2022	2
Hubbs-2249-Tt	NA	209	Female	Juvenile	28.14325	-80.60836	Rainy	9/2/2022	3
Hubbs-2310-Tt	NA	262	Male	Adult	28.50485	-80.59888	Dry	1/23/2023	3
Hubbs-2322-Tt	*Dolphin 9*	160	Female	Calf	28.07693	-80.6013	Rainy	6/13/2023	2

Hubbs-2327-Tt	NA	252	Male	Adult	28.07769	-80.60023	Rainy	6/30/2023	3
Hubbs-2335-Tt	NA	238	Female	Adult	28.40687	-80.71009	Rainy	7/30/2023	3
Hubbs-2341-Tt	NA	274	Female	Adult	28.27679	-80.6052	Rainy	10/2/2023	2
Hubbs-2348-Tt	*Dolphin 10*	110	Female	Calf	28.36887	-80.6838	Rainy	11/26/2023	2
Hubbs-2401-Tt	NA	229	Female	Juvenile	28.33205	-80.6959	Dry	1/1/2024	2
Hubbs-2402-Tt	NA	152	Female	Calf	28.23449	-80.6743	Dry	1/4/2024	2
Hubbs-2403-Tt	NA	217	Male	Juvenile	28.32448	-80.6923	Dry	1/4/2024	3

Table S2. Raw data for IRL common bottlenose dolphins (n = 156) evaluated for MC exposure (2005-2024), including sample type tested, MC values, and liver histopathology results (NA = data or samples not available, MDL = minimum detection limit, MMPB = 2-methyl-3-methoxy-4-phenylbutyric acid technique, NSF = no significant histopathological findings).

Sender ID	Publication ID	Sample type for MC ELISA	MC ELISA level (ng/g)	MC MMPB level	Liver histopathology
HBOI-1303-Tt	NA	Liver	< MDL	NA	NSF
HBOI-1309-Tt	NA	Liver	< MDL	NA	NSF
HBOI-1409-Tt	NA	Liver	< MDL	NA	NSF
HBOI-1601-Tt	NA	Liver	< MDL	< MDL	Hepatitis (Lymphoplasmacytic, Periportal, Mild, Chronic); Fibroplasia; Pigment (Multifocal, Mild, Chronic)
HBOI-1602-Tt	NA	Liver	< MDL	NA	NSF
HBOI-2101-Tt	NA	Feces<br>Liver	< MDL<br>< MDL	NA	NSF
HBOI-2201-Tt	NA	Feces<br>Liver	< MDL<br>< MDL	NA	Hepatitis (Neutrophilic, Fibrinous, Mild, Acute); Bacteria
HBOI-2203-Tt	NA	Liver	< MDL	NA	Bacteria; Hemorrhage (Mild)
HBOI-2205-Tt	NA	Liver	< MDL	< MDL	EMH; Atrophy (Mild)
Hubbs-0510-Tt	NA	Liver	< MDL	NA	NSF
Hubbs-0517-Tt	*Dolphin 1*	Liver	6.7	< MDL	NSF
Hubbs-0523-Tt	NA	Liver	< MDL	NA	NSF
Hubbs-0541-Tt	NA	Liver	< MDL	< MDL	Degenerative Hepatopathy (Diffuse, Mild)
Hubbs-0630-Tt	NA	Liver	< MDL	NA	NSF
Hubbs-0701-Tt	NA	Liver	< MDL	NA	NA
Hubbs-0709-Tt	*Dolphin 2*	Liver	6.8	< MDL	NA
Hubbs-0717-Tt	NA	Liver	< MDL	< MDL	Fibrosis (Periportal, Capsular, Multifocal, Moderate, Chronic); Congestion (Diffuse, Mild, Acute, Centrilobular); Hemosiderosis (Diffuse, Mild)
Hubbs-0723-Tt	NA	Liver	< MDL	NA	NA
Hubbs-0760-Tt	NA	Liver	< MDL	NA	NSF
Hubbs-0805-Tt	NA	Liver	< MDL	NA	Hepatitis (Necrotic, Multifocal, Mild)
Hubbs-0808-Tt	NA	Liver	< MDL	NA	NSF
Hubbs-0825-Tt	NA	Liver	< MDL	NA	NSF
Hubbs-0827-Tt	*Dolphin 3*	Liver	34.2	< MDL	NA
Hubbs-0828-Tt	NA	Liver	< MDL	< MDL	Degenerative Hepatopathy (Multifocal, Mild, Acute)

Hubbs-0831-Tt	*Dolphin 4*	Liver	4.4	< MDL	NSF
Hubbs-0837-Tt	NA	Liver	< MDL	NA	NSF
Hubbs-0838-Tt	NA	Liver	< MDL	NA	NSF
Hubbs-0848-Tt	NA	Liver	< MDL	NA	NSF
Hubbs-0854-Tt	NA	Liver	< MDL	NA	Hemosiderosis (Multifocal, Mild)
Hubbs-0875-Tt	NA	Liver	< MDL	< MDL	Fibrosis (Periportal, Capsular, Multifocal, Mild, Chronic)
Hubbs-0901-Tt	NA	Liver	< MDL	NA	NSF
Hubbs-0910-Tt	NA	Liver	< MDL	NA	Congestion (Centrilobular, Moderate, Diffuse)
Hubbs-0915-Tt	NA	Liver	< MDL	< MDL	Fibrosis (Hepatic, Mild-Moderate, Chronic); Hepatitis (Mononuclear, Multifocal, Mild)
Hubbs-0924-Tt	NA	Liver	< MDL	< MDL	Hemosiderosis (Mild-Moderate, Diffuse); Fibrosis (Hepatic, Moderate, Chronic)
Hubbs-0940-Tt	NA	Liver	< MDL	< MDL	Hepatitis (Diffuse, Mild-Moderate); Necrosis; Degeneration
Hubbs-0942-Tt	NA	Liver	< MDL	NA	NSF
Hubbs-0955-Tt	NA	Liver	< MDL	NA	NA
Hubbs-1045-Tt	NA	Liver	< MDL	NA	NSF
Hubbs-1071-Tt	NA	Liver	< MDL	NA	NSF
Hubbs-1073-Tt	NA	Liver	< MDL	NA	NSF
Hubbs-1107-Tt	NA	Liver	< MDL	NA	NSF
Hubbs-1122-Tt	NA	Liver	< MDL	< MDL	Congestion (Multifocal, Mild-Moderate); Vacuolation; Fibrosis (Periportal, Mild)
Hubbs-1132-Tt	NA	Liver	< MDL	NA	NA
Hubbs-1202-Tt	NA	Liver	< MDL	< MDL	Fibrosis (Periportal, Multifocal, Mild)
Hubbs-1212-Tt	NA	Liver	< MDL	NA	Fibrosis (Periportal, Moderate, Chronic)
Hubbs-1216-Tt	NA	Liver	< MDL	NA	Hepatitis (Mild, Multifocal)
Hubbs-1217-Tt	*Dolphin 5*	Liver	34.2	< MDL	Lipidosis (Mild-Moderate); Dysfunction
Hubbs-1227-Tt	NA	Liver	< MDL	NA	NSF
Hubbs-1235-Tt	NA	Liver	< MDL	NA	NA
Hubbs-1249-Tt	NA	Liver	< MDL	NA	NA
Hubbs-1255-Tt	NA	Liver	< MDL	NA	NA
Hubbs-1307-Tt	NA	Liver	< MDL	< MDL	Lipidosis (Diffuse, Moderate-Severe)
Hubbs-13102-Tt	*Dolphin 6*	Liver	3.3	< MDL	NSF

Hubbs-13104-Tt	NA	Liver	< MDL	< MDL	Fibrosis (Portal, Multifocal, Mild); Hepatitis (Lymphoplasmacytic, Neutrophilic, Periportal, Mild); EMH (Multifocal, Mild)
Hubbs-13105-Tt	NA	Liver	< MDL	NA	Hyperplasia (Bile Duct, Multifocal, Mild); Fibrosis (Portal, Mild)
Hubbs-13107-Tt	NA	Liver	< MDL	NA	NSF
Hubbs-1310-Tt	NA	Liver	< MDL	< MDL	Hemosiderosis (Mild, Diffuse); Hepatitis (Mild, Multifocal)
Hubbs-1312-Tt	NA	Liver	< MDL	NA	Hepatitis (Histiocytic, Diffuse, Mild)
Hubbs-1315-Tt	NA	Liver	< MDL	NA	NSF
Hubbs-1319-Tt	NA	Liver	< MDL	< MDL	EMH (Multifocal, Mild); Hemosiderosis (Diffuse)
Hubbs-1328-Tt	NA	Liver	< MDL	< MDL	Fibrosis (Portal, Capsular, Diffuse, Moderate)
Hubbs-1331-Tt	NA	Liver	< MDL	NA	Hemosiderosis
Hubbs-1332-Tt	NA	Liver	< MDL	< MDL	Hepatitis (Lymphoplasmacytic, Multifocal, Mild-Moderate, Chronic); Lipidosis (Periportal, Multifocal, Mild)
Hubbs-1333-Tt	NA	Liver	< MDL	< MDL	Hepatitis (Lymphoplasmacytic, Multifocal, Mild, Chronic); Hemosiderosis
Hubbs-1334-Tt	NA	Liver	< MDL	< MDL	Fibrosis (Periportal, Mild, Chronic)
Hubbs-1351-Tt	NA	Liver	< MDL	NA	NSF
Hubbs-1352-Tt	NA	Liver	< MDL	< MDL	Fibrosis (Periportal)
Hubbs-1353-Tt	*Dolphin 7*	Liver	26.2	< MDL	Lipidosis (Diffuse, Mild)
Hubbs-1355-Tt	NA	Liver	< MDL	NA	Hemosiderosis (Mild)
Hubbs-1371-Tt	NA	Liver	< MDL	NA	NSF
Hubbs-1383-Tt	NA	Liver	< MDL	< MDL	Fibrosis (Capsular, Periportal, Diffuse, Mild)
Hubbs-1387-Tt	NA	Liver	< MDL	NA	NSF
Hubbs-1408-Tt	NA	Liver	< MDL	NA	NSF
Hubbs-1416-Tt	NA	Liver	< MDL	NA	NA
Hubbs-1431-Tt	NA	Liver	< MDL	NA	Necrosis (Centrilobular); Congestion (Acute, Passive, Diffuse, Moderate); Fibrosis (Portal, Multifocal, Moderate-Marked); Hepatitis (Lymphoplasmacytic, Portal)
Hubbs-1433-Tt	NA	Liver	< MDL	< MDL	Lipidosis (Mild)/Vacuolar Hepatopathy
Hubbs-1440-Tt	NA	Liver	< MDL	< MDL	Lipidosis (Diffuse, Marked)
Hubbs-1448-Tt	NA	Liver	< MDL	NA	Fibrosis (Portal, Diffuse, Mild-Moderate); Hyperplasia (Biliary); Hepatitis (Lymphoplasmacytic, Periportal)
Hubbs-1450-Tt	NA	Liver	< MDL	< MDL	Hepatitis (Suppurative, Multifocal-Coalescing, Marked); EMH (Diffuse, Moderate)

Hubbs-1451-Tt	NA	Liver	< MDL	< MDL	Hyperplasia (Biliary); Fibrosis (Portal, Multifocal, Mild & Capsular, Focally Extensive); Vacuolation (Multifocal, Mild)
Hubbs-1466-Tt	NA	Liver	< MDL	NA	Hepatitis (Necrosuppurative, Histiocytic, Multifocal-Coalescing, Moderate-Marked); Syncytial Cells; Intranuclear Viral Inclusions; Lipidosis (Diffuse, Moderate); EMH (Multifocal, Mild)
Hubbs-1468-Tt	NA	Liver	< MDL	NA	NSF
Hubbs-1486-Tt	*Dolphin 11*	Liver	< MDL	NA	Necrosis (Centrilobular & Midzonal to Submassive, Multifocal-Coalescing, Marked); Hemorrhage; Infarcts (Acute); Dysfunction
Hubbs-1491-Tt	NA	Liver	< MDL	NA	NSF
Hubbs-1496-Tt	NA	Liver	< MDL	NA	NSF
Hubbs-1501-Tt	NA	Liver	< MDL	< MDL	Fibrosis (Portal, Multifocal, Mild); Hepatitis (Lymphoplasmacytic, Periportal)
Hubbs-1506-Tt	NA	Liver	< MDL	NA	NSF
Hubbs-1508-Tt	*Dolphin 8*	Liver	6.8	< MDL	NA
Hubbs-1509-Tt	NA	Liver	< MDL	< MDL	Lipidosis (Moderate)
Hubbs-1520-Tt	NA	Liver	< MDL	NA	NSF
Hubbs-1523-Tt	NA	Liver	< MDL	NA	NSF
Hubbs-1525-Tt	NA	Liver	< MDL	NA	NSF
Hubbs-1544-Tt	NA	Liver	< MDL	NA	NSF
Hubbs-1549-Tt	NA	Liver	< MDL	< MDL	Fibrosis (Portal, Diffuse, Mild-Moderate); Hyperplasia (Biliary, Moderate); Hepatitis (Lymphoplasmacytic, Periportal)
Hubbs-1603-Tt	NA	Liver	< MDL	NA	NSF
Hubbs-1607-Tt	NA	Liver	< MDL	NA	NSF
Hubbs-1617-Tt	NA	Liver	< MDL	NA	NSF
Hubbs-1656-Tt	NA	Liver	< MDL	NA	Congestion (Chronic, Passive)
Hubbs-1659-Tt	NA	Liver	< MDL	NA	NA
Hubbs-1666-Tt	*Dolphin 12*	Liver	< MDL	< MDL	Fibrosis (Portal, Diffuse, Moderate, Bile duct); Hyperplasia (Bile Duct); Hepatitis (Lymphoplasmacytic, Periportal, Patchy); Ductitis (Lymphoplasmacytic, Mild); Dysfunction; Cause of Stranding
Hubbs-1728-Tt	NA	Liver	< MDL	NA	NSF
Hubbs-1730-Tt	NA	Liver	< MDL	NA	NA

Hubbs-1743-Tt	NA	Liver	< MDL	< MDL	Hepatitis (Lymphoplasmacytic, Periportal & Granulomatous, Periportal, Focal); Fibrosis (Portal, Multifocal, Moderate); Hyperplasia (Biliary)
Hubbs-1801-Tt	NA	Liver	< MDL	< MDL	Hepatitis (Lymphoplasmacytic, Focal, Mild)
Hubbs-1802-Tt	NA	Liver	< MDL	NA	NSF
Hubbs-1804-Tt	NA	Liver	< MDL	< MDL	Fibrosis (Capsular, Diffuse, Moderate & Portal, Multifocal, Mild)
Hubbs-1805-Tt	NA	Liver	< MDL	< MDL	Fibrosis (Portal, Diffuse, Mild-Moderate); Hyperplasia (Biliary, Mild); Hepatitis (Lymphoplasmacytic, Periportal)
Hubbs-1812-Tt	NA	Liver	< MDL	NA	NA
Hubbs-1815-Tt	NA	Liver	< MDL	NA	NSF
Hubbs-1830-Tt	NA	Liver	< MDL	NA	Hepatitis (Fibronecrotic, Neutrophilic, Multifocal-Coalescing, Moderate, Subacute)
Hubbs-1840-Tt	NA	Liver	< MDL	NA	NA
Hubbs-1844-Tt	NA	Liver	< MDL	NA	NSF
Hubbs-1848-Tt	NA	Liver	< MDL	NA	NA
Hubbs-1903-Tt	NA	Liver	< MDL	NA	NSF
Hubbs-1904-Tt	NA	Liver	< MDL	NA	Necrosis (Coagulative, Patchy, Mild)
Hubbs-1911-Tt	NA	Liver	< MDL	< MDL	Fibrosis (Hepatic, Mild-Moderate); Hepatitis (Necrotic, Histiocytic, Multifocal, Mild)
Hubbs-1914-Tt	NA	Liver	< MDL	< MDL	Hepatitis (Periportal, Lymphoplasmacytic, Multifocal, Mild)
Hubbs-1919-Tt	NA	Liver	< MDL	NA	NSF
Hubbs-1934-Tt	NA	Liver	< MDL	< MDL	Necrosis (Multifocal, Mild)
Hubbs-2003-Tt	NA	Liver	< MDL	NA	NSF
Hubbs-2004-Tt	NA	Liver	< MDL	NA	Atrophy (Centrilobular); Lipidosis; Hepatitis (Neutrophilic, Histiocytic, Multifocal, Mild, Subacute); Amyloidosis (Hepatic Artery)
Hubbs-2018-Tt	NA	Liver	< MDL	< MDL	EMH; Fibrosis (Portal, Multifocal, Mild); Hepatitis (Neutrophilic, Portal)
Hubbs-2025-Tt	NA	Liver	< MDL	NA	Hepatitis (Necrosuppurative, Multifocal, Marked-Severe)
Hubbs-2046-Tt	NA	Liver	< MDL	NA	NA
Hubbs-2048-Tt	*Dolphin 13*	Liver	< MDL	< MDL	Hepatitis (Portal, Lymphoplasmacytic, Neutrophilic, Multifocal, Mild, Chronic-Active); Atrophy (Multifocal, Mild); Fibrosis (Portal); Dysfunction; Cause of Death
Hubbs-2102-Tt	NA	Liver	< MDL	NA	Infarct (Hemorrhagic, Acute)
Hubbs-2104-Tt	NA	Liver	< MDL	NA	NSF

Hubbs-2105-Tt	NA	Liver	< MDL	NA	NSF
Hubbs-2115-Tt	NA	Liver	< MDL	NA	NSF
Hubbs-2117-Tt	NA	Resp vapor plate	< MDL*	NA	NA
Hubbs-2121-Tt	NA	Liver	< MDL	< MDL	Necrosis (Focal, Centrilobular)
Hubbs-2127-Tt	NA	Liver	< MDL	< MDL	Lipidosis (Diffuse)
Hubbs-2128-Tt	NA	Liver	< MDL	< MDL	Fibrosis (Portal, Multifocal, Mild-Moderate); Hyperplasia (Biliary, Mild)
Hubbs-2129-Tt	NA	Liver	< MDL	NA	NSF
Hubbs-2133-Tt	NA	Liver	< MDL	< MDL	Fibrosis (Focal); Hyperplasia (Biliary)
Hubbs-2146-Tt	NA	Liver	< MDL	< MDL	Fibrosis (Portal, Multifocal-Coalescing, Mild-Moderate)
Hubbs-2148-Tt	NA	Liver	< MDL	NA	Necrosis
Hubbs-2206-Tt	NA	Liver	< MDL	< MDL	Vacuolation (Periportal-Midzonal, Discrete)
Hubbs-2207-Tt	NA	Liver	< MDL	< MDL	Lipidosis (Moderate, Diffuse); Hepatitis (Necrosuppurative, Focal, Mild)
Hubbs-2211-Tt	NA	Liver	< MDL	< MDL	Fibrosis (Portal, Multifocal, Mild-Moderate); Hyperplasia (Biliary); Hepatitis (Lymphoplasmacytic, Portal); Myeloid Leukemia (Eosinophilic, Focal)
Hubbs-2214-Tt	NA	Liver	< MDL	< MDL	Congestion (Centrilobular, Mild-Moderate); Fibrosis (Periportal, Moderate); Hyperplasia (Biliary); Lipidosis (Moderate)
Hubbs-2216-Tt	NA	Liver	< MDL	NA	NSF
Hubbs-2224-Tt	NA	Liver	< MDL	NA	Atrophy (Diffuse, Mild)
Hubbs-2225-Tt	NA	Liver	< MDL	NA	NSF
Hubbs-2236-Tt	NA	Liver	< MDL	NA	Hepatitis (Necrosuppurative, Multifocal, Moderate)
Hubbs-2238-Tt	NA	Liver	< MDL	NA	NA
Hubbs-2249-Tt	NA	Liver	< MDL	NA	NSF
Hubbs-2310-Tt	NA	Liver<br>Resp swab	< MDL<br>1.991*	NA	NSF
Hubbs-2322-Tt	*Dolphin 9*	Feces<br>Liver<br>Resp swab	2.3<br>< MDL<br>0.948*	NA	NSF
Hubbs-2327-Tt	NA	Liver	< MDL	< MDL	Fibrosis (Portal, Multifocal, Mild); Hyperplasia (Biliary, Mild)
Hubbs-2335-Tt	NA	Liver	< MDL	NA	NSF
Hubbs-2341-Tt	NA	Feces<br>Liver<br>Resp vapor plate	< MDL<br>< MDL<br>< MDL*	NA	Hepatitis/Cholangitis (Lymphoplasmacytic to Granulomatous, Marked); Intralesional Trematode and Ova, Bile Duct Ectasia (Regionally Extensive, Moderate)

Hubbs-2348-Tt	*Dolphin 10*	Feces<br>Liver	3<br>< MDL	NA	NA
Hubbs-2401-Tt	NA	Liver<br>Resp swab	< MDL<br>1.675*	NA	NA
Hubbs-2402-Tt	NA	Feces<br>Liver	< MDL<br>< MDL	NA	NA
Hubbs-2403-Tt	NA	Liver<br>Resp swab	< MDL<br>1.935*	NA	NA

*Samples did not undergo SPE cleanup

Table S3. The actual MC level versus the recovered concentration via ADDA-ELISA of spiked sterile respiratory vapor petri dish samples. Respiratory vapor is under evaluation as a non-invasive MC detection method in free-ranging bottlenose dolphins.

**Spike**	**Actual MC level (ng/ml)**	**ELISA MC level (ng/ml)**
Standard 0	0	< MDL
Standard 1	0.03	< MDL
Standard 2	0.08	< MDL
Standard 3	0.2	< MDL
Standard 4	0.4	0.23
Standard 5	1	0.83
Control	0.15	0.21
Blank	0	0.22

Table S4. The actual MC level versus the recovered concentration via ADDA-ELISA of spiked sterile cotton tip swabs. Swabs of respiratory fluid/vapor are under evaluation as a non-invasive MC detection method in free-ranging bottlenose dolphins.

Spike	Actual MC level (ng/ml)	ELISA MC level (ng/ml)
Standard 0	0	0.18
Standard 1	0.03	0.25
Standard 2	0.08	0.29
Standard 3	0.2	0.38
Standard 4	0.4	0.53
Standard 5	1	1.18
Control	0.15	0.29
Blank	0	0.18
